# Contributing to Elimination of Cross-Border Malaria Through a Standardized Solution for Case Surveillance, Data Sharing, and Data Interpretation: Development of a Cross-Border Monitoring System

**DOI:** 10.2196/15409

**Published:** 2020-09-01

**Authors:** Raphael Saldanha, Émilie Mosnier, Christovam Barcellos, Aurel Carbunar, Christophe Charron, Jean-Christophe Desconnets, Basma Guarmit, Margarete Do Socorro Mendonça Gomes, Théophile Mandon, Anapaula Martins Mendes, Paulo César Peiter, Lise Musset, Alice Sanna, Benoît Van Gastel, Emmanuel Roux

**Affiliations:** 1 Laboratório de Informação em Saúde Instituto de Comunicação e Informação Científica e Tecnológica em Saúde Fundação Oswaldo Cruz Rio de Janeiro Brazil; 2 Laboratoire Mixte International Sentinela Fundação Oswaldo Cruz, Universidade de Brasília, Institut de Recherche pour le Développement Rio de Janeiro Brazil; 3 Service des Centres Délocalisés de Prévention et de Soins Centre Hospitalier de Cayenne Cayenne French Guiana; 4 Sciences Économiques et Sociales de la Santé et Traitement de l'Information Médicale Aix Marseille Université, Institut National de la Santé et de la Recherche Médicale, Institut de Recherche pour le Développement Marseille France; 5 Espace-Dev Institut de Recherche pour le Développement Université de Montpellier, Université de La Réunion, Université de Guyane, Université des Antilles Cayenne, French Guiana, and Montpellier France; 6 Superintendência de Vigilância em Saúde do Estado do Amapá Macapá Brazil; 7 Universidade Federal do Amapá Oiapoque Brazil; 8 Laboratório de Doenças Parasitárias Instituto Oswaldo Cruz Fundação Oswaldo Cruz Rio de Janeiro Brazil; 9 Laboratoire de Parasitologie Institut Pasteur de la Guyane Cayenne French Guiana; 10 Centre National de Référence du Paludisme Pôle Zones Endémiques Françaises World Health Organization Collaborating Center for Surveillance of Antimalarial Drug Resistance Cayenne French Guiana; 11 Agence Régionale de Santé de Guyane Cayenne French Guiana

**Keywords:** cross-border malaria, surveillance, data interoperability, data visualization, French Guiana, Brazil

## Abstract

**Background:**

Cross-border malaria is a significant obstacle to achieving malaria control and elimination worldwide.

**Objective:**

This study aimed to build a cross-border surveillance system that can make comparable and qualified data available to all parties involved in malaria control between French Guiana and Brazil.

**Methods:**

Data reconciliation rules based on expert knowledge were defined and applied to the heterogeneous data provided by the existing malaria surveillance systems of both countries. Visualization dashboards were designed to facilitate progressive data exploration, analysis, and interpretation. Dedicated advanced open source and robust software solutions were chosen to facilitate solution sharing and reuse.

**Results:**

A database gathering the harmonized data on cross-border malaria epidemiology is updated monthly with new individual malaria cases from both countries. Online dashboards permit a progressive and user-friendly visualization of raw data and epidemiological indicators, in the form of time series, maps, and data quality indexes. The monitoring system was shown to be able to identify changes in time series that are related to control actions, as well as differentiated changes according to space and to population subgroups.

**Conclusions:**

This cross-border monitoring tool could help produce new scientific evidence on cross-border malaria dynamics, implementing cross-border cooperation for malaria control and elimination, and can be quickly adapted to other cross-border contexts.

## Introduction

The Global Technical Strategy of the World Health Organization (WHO) [1] aims for a 90% reduction in global malaria mortality and incidence by 2030 in comparison with 2015 levels, notably by “transforming malaria surveillance into a core intervention.”

However, several obstacles make such a strategy difficult to apply and the elimination target challenging to reach. One of them is *cross-border malaria* [2-7]. Cross-border malaria does not only refer to the malaria cases that cross international borders, but also to all aspects of the disease within cross-border living territories that require actual cross-border visions. However, from one country to another, differences are observed in disease diagnosis and treatment protocols, the epidemiological information collected, database structures, information representations (ie, database attribute names, formats, encoding, etc), data access protocols and rights, and so forth. Such differences prevent the border countries from having a shared and unified view of the cross-border epidemiological situation and, thus, to jointly design and implement efficient control actions. Cross-border epidemiological surveillance systems are required to overcome such obstacles. One solution is to build them into existing national systems, when they exist, by ensuring data interoperability. However, data reconciliation implies dealing with semantic, structural, and syntactic heterogeneities. Moreover, the diversity of recipients of the harmonized data (ie, health actors, health and territory managers, the general public, etc) challenges the actual and advantageous dissemination of cross-border harmonized data and knowledge. In fact, the potential recipients differ notably in their objectives, background knowledge on the disease, technological skills, and languages.

The French Guiana–Brazil border is an endemic malaria region [8]. The Franco-Brazilian cooperation agreement of May 28, 1996, led to the creation of the Joint Commission for Cross-Border Cooperation between French Guiana and Brazil. A subworking group has been working exclusively on health-related issues since 2009. Notably, this resulted in regular epidemiological data exchanges on malaria between French Guianese and Brazilian malaria surveillance authorities. However, differences in data formats, update frequencies, spatial and temporal aggregation units, and nature of information; the lack of contextual information (ie, metadata) and shared frame of reference, notably, a cartographic representation; as well as the limited numbers of recipients of the information on both sides of the border make such a procedure inefficient in providing a unified vision of the malaria situation in the cross-border area. This consequently prevents the design and implementation of concerted control and elimination actions.

In this context, building a cross-border malaria information system (CBMIS) is needed. This requires specifying easily reproducible methods based on explicit data harmonization rules, free technological solutions, as well as information representation and dissemination good practices. Moreover, data visualization solutions for health actors, health and territory managers, and the general public are necessary to facilitate data and knowledge dissemination. This paper addresses such issues by describing a cross-border system for data harmonization and visualization implemented between French Guiana and Brazil.

## Methods

### Study Area

**Figure 1:**
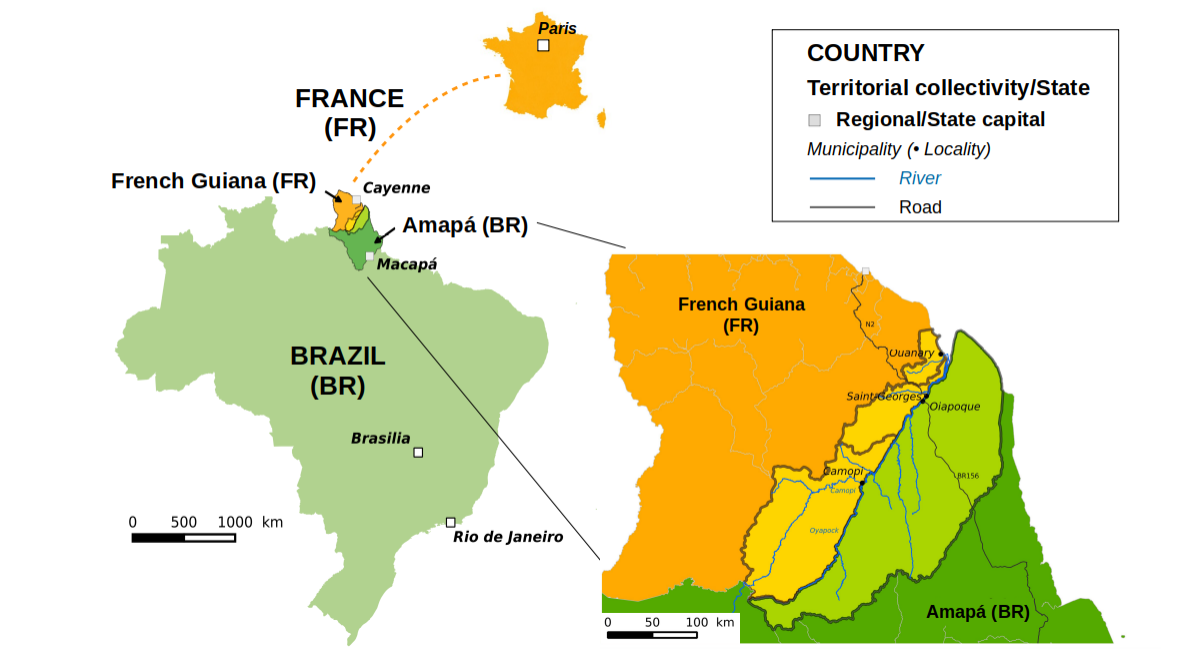
Cross-border area delimitation and administrative structuration of the region.

French Guiana—83,534 km^2^ in area with an estimated 290,691 inhabitants in 2020 [9]—is a French overseas region located in the Amazon, South America. French Guiana consists of 22 municipalities, with four of them bordering Brazil: Maripasoula, Camopi, Saint-Georges de l’Oyapock (hereafter referred to as Saint-Georges), and Ouanary. Amapá—142,829 km^2^ in area with an estimated 845,731 inhabitants in 2019 [10]—is one of the 27 states, including the federal district, of the Federative Republic of Brazil. The Amapá state is located in the Brazilian Amazon, bordering French Guiana to the north (see Figure 1).

For the development of the CBMIS, the cross-border area between French Guiana and Brazil was defined by the border municipalities of both countries, which define a coherent and continuous living territory for local populations (see Figure 1): for French Guiana, this includes Ouanary, Saint-Georges, and Camopi, with 201, 4220, and 1828 inhabitants in 2017, respectively [9]; for Brazil, this includes Oiapoque, with 27,270 inhabitants in 2019 [10]. The population living in this area is distributed over two main urban centers, Saint-Georges and Oiapoque, as well as in villages mainly located along the Oiapoque River, along the BR-156 road in Amapá, and in territories with restricted access (ie, natural parks on both sides of the border and the Brazilian Amerindian Territories).

### Data Sources and Definition of Cross-Border Malaria Cases

Concerning French Guiana, anonymized information regarding *individual malaria cases* is collected monthly from the surveillance system of the delocalized Centers for Prevention and Care (Centres Délocalisés de Prévention et de Soins [CDPSs]) operated by the Cayenne Hospital, which has been operating since 2007. Four CDPSs are present in the cross-border area: in Ouanary, Saint-Georges, Camopi, and Trois-Sauts (Camopi municipality). In this system, a malaria case is defined as any positive rapid diagnostic test (RDT) (SD BIOLINE Malaria Ag Pf/Pan in French Guiana). Such tests only distinguish *P falciparum* and non-*P falciparum* species. *New attacks* of malaria (ie, new infections due to new mosquito bites, to be distinguished from malaria notifications related to the follow-up of patients, treatment failures, or *P vivax* relapses) are not explicitly identified in the database. Each patient in the database is identified by a unique coded identifier.

Regarding Brazil, information on *individual malaria cases* is provided by the Malaria Epidemiological Surveillance Information System (Sistema de Informações de Vigilância Epidemiológica da Malaria [SIVEP-Malária]), operated by the information technology department of the unified health system (Departamento de Informática do Sistema Único de Saúde) of the Brazilian Ministry of Health. Brazil mainly uses thick smear microscopy, allowing for the identification of all *Plasmodium* species and development stages, but also the RDT (SD BIOLINE Ag Pf/Pf/Pv).

In the Brazilian database, malaria attacks related to follow-up consultations, treatment failures, and relapses are all referred to as *treatment verification slides* (lâminas de verificação de cura [LVCs]). A malaria case is considered as an LVC for *P vivax* (or for *P falciparum*) if the patient received treatment against *P vivax* (or for *P falciparum*) within the last 60 days (40 days for *P falciparum*) [11]. A non-LVC case is considered a *new case*. Patients are not identified by a unique coded identifier. The SIVEP-Malária supplies anonymized data on a monthly basis to the CBMIS through a partnership with the Oswaldo Cruz Foundation (Fundação Oswaldo Cruz [Fiocruz]). Database fields of the French and Brazilian surveillance systems that were considered in the CBMIS are detailed in Multimedia Appendix 1, Table S1.

A *cross-border malaria case* was defined as any malaria case as defined by the national surveillance systems and that was associated with (1) a notification center, (2) a patient’s residential address, *or* (3) a possible transmission location, *located in the previously defined cross-border area*.

The two surveillance systems report on the locations of notification centers, residences, or putative contamination locations, with respect to predefined and scalable lists of *localities* (ie, a locality being either isolated but inhabited places, villages, or urban neighborhoods), but without systematically providing their geographical coordinates [12]. Thus, geographical coordinates of localities were obtained through various sources: knowledge of the researchers and partners involved in the project; OpenStreetMap collaborative project; National Indigenous Foundation (for Brazilian Amerindian villages); Google and Bing satellite imagery; and Sentinel-2 satellite images from the European Space Agency, retrieved from the operating platform (Plateforme d'Exploitation des Produits Sentinel) of the Sentinel products developed by the French space agency (Centre National d’Études Spatiales).

### Data Harmonization System

**Figure 2:**
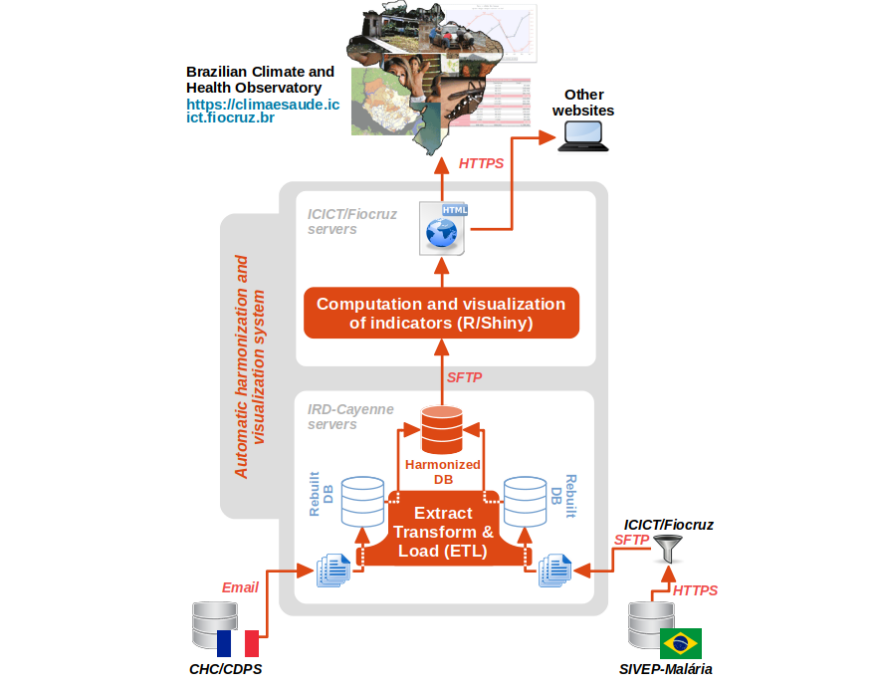
Overall system architecture and data and information flow. CDPS: Service des Centres Délocalisés de Prévention et de Soins (Department of the Centers for Prevention and Care); CHC: Centre Hospitalier de Cayenne (Cayenne Hospital); DB: database; Fiocruz: Fundação Oswaldo Cruz (Oswaldo Cruz Foundation); HTTPS: hypertext transfer protocol secure; ICICT: Instituto de Comunicação e Informação Científica e Tecnológica em Saúde (Institute of Scientific and Technological Communication and Information in Health); IRD: Institut de Recherche pour le Développement (French National Research Institute for Sustainable Development); SFTP: secure shell file transfer protocol; SIVEP-Malária: Sistema de Informações de Vigilância Epidemiológica da Malária (Malaria Epidemiological Surveillance Information System).

Harmonization was aimed at transforming the data from the two national information systems in order to make them satisfy a common harmonized data model; see Figure 2 for a representation of the global data flow, with the main harmonization steps and the data transfer protocols used.

This common harmonized data model relied, as much as possible, on existing standards: international standards or, if not available, national ones or even de facto normative representations, due to their extensive and consensual use in the knowledge areas involved in the study. In practice, harmonization consisted of changes in data types (eg, conversion from string type to integer type for the sex field in the SIVEP-Malária database), unit conversions (eg, patient age conversion from days or months to years), and data transformations that required more deep knowledge on malaria surveillance and parasitology, especially regarding *Plasmodium* species specification and new malaria case detection. The information provided by the RDT on *Plasmodium* species was more general and was the only information shared by both countries. In the harmonized database, *Plasmodium* species were consequently coded as “*P falciparum,*” “non-*P falciparum,*” “mixed infection with *P falciparum,*” or “Unspecified” (see Multimedia Appendix 1, Table S2, for details). Eventually, a *new attack* was defined in the CBMIS: for data from the SIVEP-Malária (Brazil), this was defined as any case notification that is *not* an LVC; for data from the CDPS database (French Guiana), this was defined as any *P vivax* (or *P falciparum*) case notification that occurs at least 91 days (41 days for *P falciparum*) after the last *new attack* of *P vivax* (or *P falciparum*). In fact, French epidemiologists consider that a *P vivax* malaria notification can be considered as a *new case* if it occurs more than 90 days after the last contamination [13]. Unique patient identifiers were used to reconstruct the patient notification history and to apply this *new case* detection rule.

The initial data representations within the national systems, the harmonized data model, and associated standards, as well as the harmonization rules, are provided in Multimedia Appendix 1, Table S1.

An *extract, transform, and load* (ETL) process, implemented by the free software Talend Open Studio for Big Data, was used to apply all the transformation rules.

### Harmonized Data Visualization and Dissemination

To deal with the previously mentioned barriers to information and knowledge dissemination, progressive access to information was implemented using the Shneiderman et al mantra [14]: “Overview first, zoom and filter, then details-on-demand.” Dashboards in three languages—Portuguese, French, and English—accessible to the users via the internet, using any updated browser on a computer or mobile device, were developed. The visualization tool has been implemented in two versions: a *general public* version, accessible without any authentication procedure but with restricted functionalities and data access, and an *expert* version, accessible through log-in and password and with full access to master harmonized data and functionalities. Multimedia Appendix 1, Table S3, details the functionalities of the two versions.

The visualization dashboards were implemented with the R package Shiny (RStudio) [15]. They were made accessible online [16,17]. Access to dashboards was also provided through the Brazilian Climate and Health Observatory [18], more precisely via the webpage dedicated to the Amapá–French Guiana *surveillance area* [19].

### Legal and Ethical Considerations

Data on malaria cases are received already anonymized from the CDPS department and the SIVEP-Malária. The CBMIS ensures the automatic processing of patient-related personal data and the transfer of these data to the Brazilian partner. This required the following: (1) the authorization from the French data protection authority (Commission Nationale de l’Informatique et des Libertés [CNIL]), which verifies compliance with the General Data Protection Regulation (EU) 2016/679 (CNIL deliberation No. 2019-025 of 28 February 2019, request for authorization No. 2135363), and (2) the ratification of the *European Union standard contractual clauses for transfers between two data controllers*. In Brazil, all the actions carried out were authorized as part of the Fiocruz public health activities, as per the Brazilian *free access* law 12.527 of November 18, 2011, and in compliance with law 13.709 of August 14, 2018.

The compliance with legal requirements demanded a specific algorithmic development for new case identification in the French Guiana database, which is detailed in Multimedia Appendix 1, Figure S1.

## Results

The CBMIS has been implemented and updated and harmonized data are delivered monthly. Data are available starting from 2003 and 2007 for the SIVEP-Malária Brazilian system and the CDPS French Guiana database, respectively. Some key harmonized database contents for the common period (ie, since 2007) are presented hereafter.

**Figure 3:**
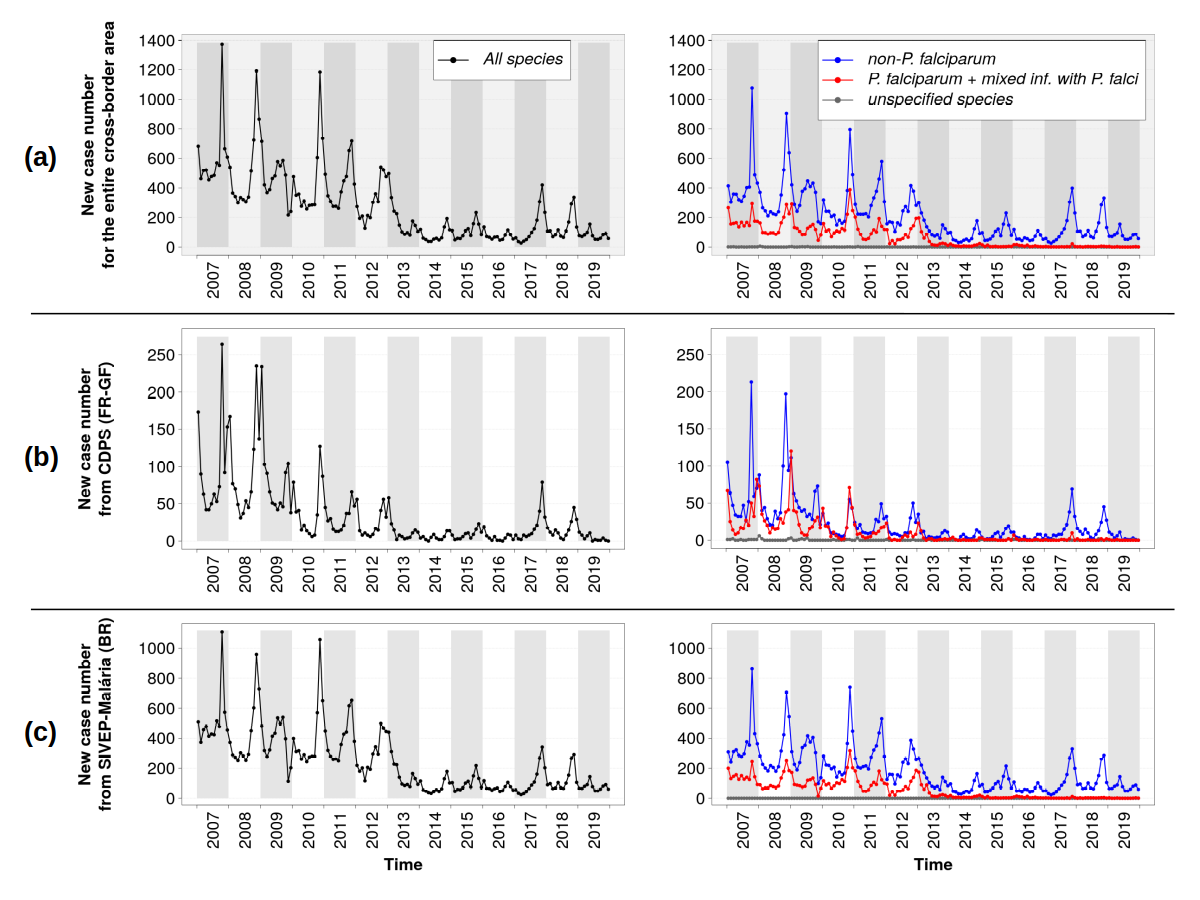
Number of new monthly malaria cases reported in the cross-border area from 2007 to 2019: (a) the cross-border area as a whole; (b) cases recorded in the database of the Department of the Centers for Prevention and Care (Service des Centres Délocalisés de Prévention et de Soins [CDPS]) in French Guiana (FR-GF); (c) cases recorded in the Malaria Epidemiological Surveillance Information System (Sistema de Informações de Vigilância Epidemiológica da Malária [SIVEP-Malária]) in Brazil (BR).

Figure 3 shows the number of new malaria cases in the cross-border area as a whole and as a function of the country of notification.

**Figure 4:**
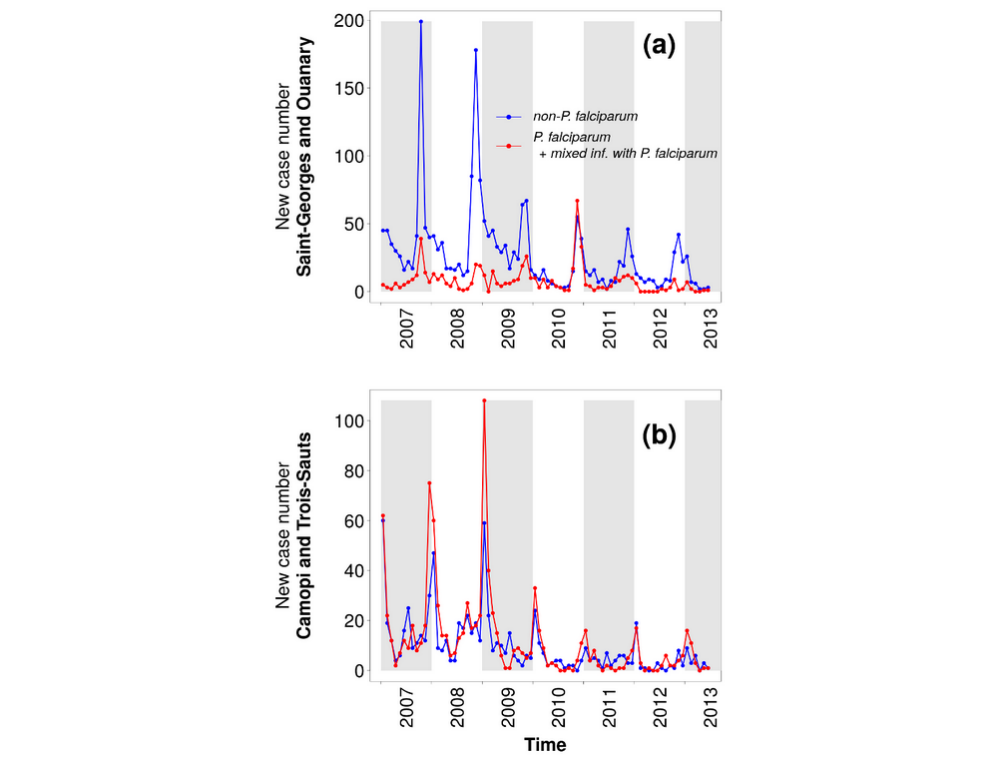
Monthly reported malaria cases by species at the Centers for Prevention and Care (Centres Délocalisés de Prévention et de Soins [CDPSs]) of (a) Saint Georges de l’Oyapock and Ouanary and (b) Camopi and Trois Sauts, between January 2007 to June 2013.

Cases notified by both countries, globally, presented comparable dynamics, with a clear seasonality showing a peak between October and December (ie, at the end of the dry season and the early beginning of the rainy season). Four main phases can be distinguished over the total period:

1. January 2007 to June 2013: high but decreasing number of cases. Figure 3 (b) shows a two-peak epidemic curve in cases notified in the CDPS database (French Guiana) for this period, except for the year 2010. These two peaks were associated with different subregions and, to a lesser extent, with different *Plasmodium* species (see Figure 4). The first peak (October to November) corresponded with the lower Oyapock River region (ie, Saint-Georges and Ouanary), with a majority of non-*P falciparum* cases, as seen in Figure 4 (a); the second peak (December to January) corresponded to the upper Oyapock River region (ie, Trois Sauts and Camopi), with a majority of *P falciparum* cases, as seen in Figure 4 (b). Moreover, two subphases can be seen during this period in the cases provided by the CDPS database: a high and quite stable number of cases in 2007 and 2008 and a significant drop in the number of cases in 2009, followed by a progressive decrease up to 2013.
2. July 2013 to December 2016: low number of cases with relative interannual stability, despite a higher number of cases in 2015. The year 2013 particularly corresponded to a significant drop in the number of *P falciparum* cases (see Figure 3).
3. January 2017 to December 2018: recrudescence of *P vivax* cases.
4. January 2019: number of cases comparable with the 2013-2016 period, even lower for CDPS data, with a peak earlier in the year in May, particularly marked in the data provided by the SIVEP-Malária (Brazil).

**Figure 5:**
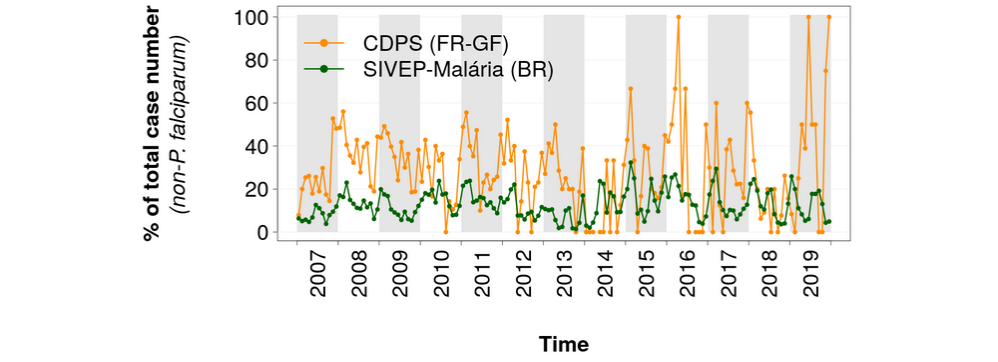
Percentages of cases associated with follow-up, treatment failures, or relapses for non-P falciparum cases in the database of the Department of the Centers for Prevention and Care (Service des Centres Délocalisés de Prévention et de Soins [CDPS]) in French Guiana (FR-GF) and the Malaria Epidemiological Surveillance Information System (Sistema de Informações de Vigilância Epidemiológica da Malária [SIVEP-Malária]) in Brazil (BR).

For non-*P falciparum* species, a significantly higher percentage of cases related to follow-up, treatment failures, and relapses were identified in the CDPS database (see Figure 5). During the whole period, the average percentages were 28.7% and 12.7% in the CDPS database and in the SIVEP-Malária, respectively. As the number of cases became very low in French Guiana in 2016 and 2019, no malaria case was reported for some months; for other months, 100% of the cases were associated with follow-ups, putative treatment failures, or relapses.

**Figure 6:**
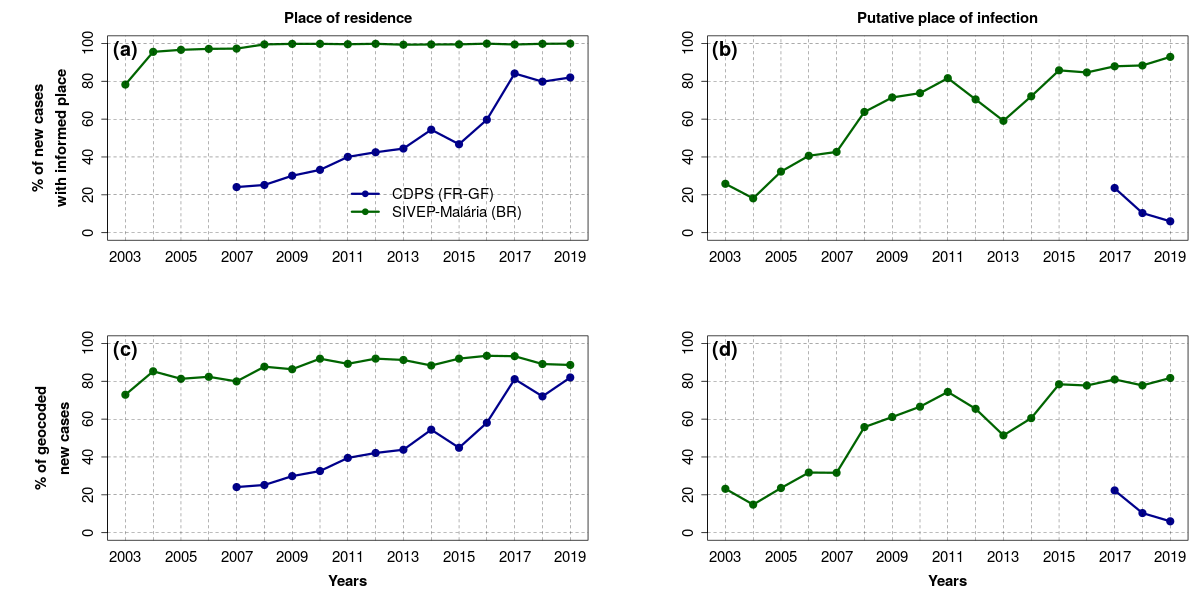
Percentage of malaria cases in the database of the Department of the Centers for Prevention and Care (Service des Centres Délocalisés de Prévention et de Soins [CDPS]) in French Guiana (FR-GF) and in the Malaria Epidemiological Surveillance Information System (Sistema de Informações de Vigilância Epidemiológica da Malária [SIVEP-Malária]) in Brazil (BR) associated with (a) a place of residence; (b) a putative place of infection; (c) a geolocalized place of residence; and (d) a geolocalized putative place of infection. Putative places of infection were not stored in the CDPS database before 2017.

In the CDPS database, the percentage of cases associated with a place of residence increased from less than 30% in 2007 to more than 80% since 2017, as seen in Figure 6 (a). On the other hand, 100% of the new cases from the SIVEP-Malária database were associated with a place of residence since 2008, as seen in Figure 6 (a).

Concerning the putative place of infection of the new cases, the information has only been stored in the CDPS database since 2017. Such information remained rare and even tended to be rarer in the CDPS database, passing from about 20% of the new cases in 2017 to less than 10% in 2019 as seen in Figure 6 (b). In the SIVEP-Malária database, such information was much more present, with more than 80% of the new cases associated with a possible place of infection since 2015 as seen in Figure 6 (b).

The specific work carried out in this study to geolocalize, or geocode, localities resulted in 100% and 52.4% of geolocalized localities of the cross-border area for the French Guiana and Brazilian sides, respectively. However, in the SIVEP-Malária, the relatively small proportion of geolocalized localities (52.4%) had little impact on the number of cases actually geolocalized, with about 90% and 80% of the cases geocoded since 2015 in relation with the places of residence and probable places of infection, respectively, as seen in Figure 6 (c) and (d).

**Figure 7:**
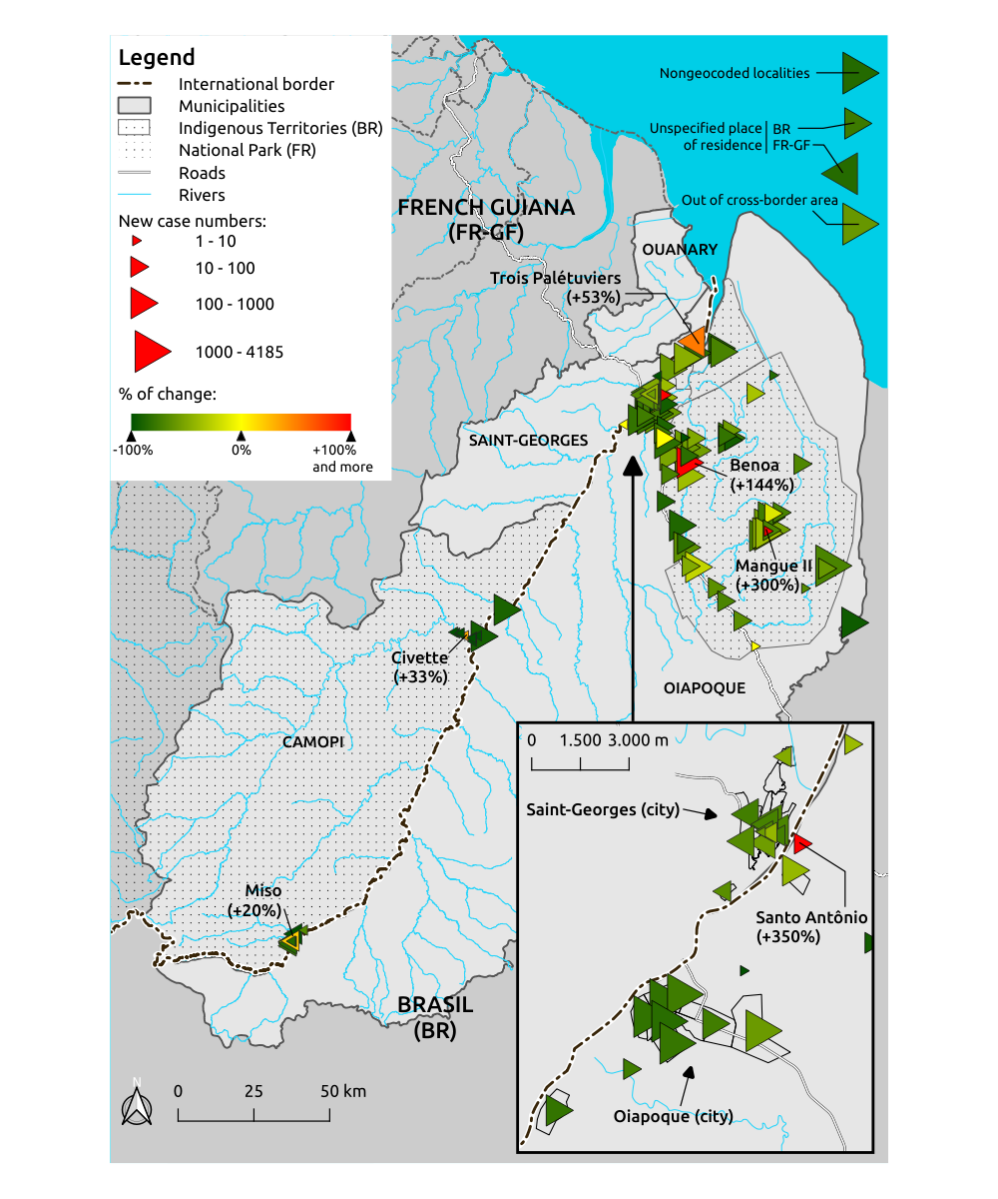
Number of reported malaria cases as a function of patients’ places of residence. Triangles with apexes oriented to the right correspond to Brazilian localities; triangles with apexes oriented to the left correspond to French localities. The triangle size is a function of the case number. The triangle color is a function of the percentage of change in the case number between the following two periods: January 2007 to June 2013 and July 2013 to December 2019.

Figure 7 shows an example of a map realized with the harmonized data of the CBMIS. It represents the numbers of new cases as a function of the places of residence of the patients, from January 2007 to December 2019, jointly with the percentage of change in the case numbers between the two main periods previously described: January 2007 to June 2013 and July 2013 to December 2019. The map shows a significant decrease in almost the entire cross-border area. The decrease was very significant in the Camopi municipality and in the urban quarters of the Oiapoque city. The decrease was significant but less important in the Amerindian communities of the Oiapoque municipality and in the Saint-Georges municipality. Some localities experienced an increase in case numbers between the two periods: the two Amerindian localities Benoa and Trois Palétuviers, in Brazil and French Guiana, respectively, had a significant increase from 34 to 84 cases (144%) and 93 to 142 cases (53%), respectively. The Amerindian locality Mangue II (Brazil), the locality Santo Antônio (Brazil), as well as the two Amerindian localities Civette and Miso in the Camopi municipality (French Guiana) experienced a nonsignificant increase in regard to the total number of cases.

## Discussion

### Principal Findings

The results showed the potential of the CBMIS for the analysis of cross-border malaria dynamics, in both space and time. Such a system also allows for pointing out similarities and differences in the epidemiological situations of both countries. As it is shown hereafter, such similarities and differences can be interpreted in terms of control strategies. In the following paragraphs, methodological aspects of the proposed approach and the previously presented results are discussed. However, specific and deep investigations of cross-border epidemiological issues are out of the scope of this paper.

### Definition of Cross-Border Malaria Cases

Human mobility is an important issue when considering border regions [2]. By differentiating between places of residence, notification, and infection, the CBMIS allows an estimation of internal and external flows in the area and facilitates the identification of autochthonous and imported malaria cases. Such differentiation also allows for conducting studies from different viewpoints, notably on environmental determinants of the transmission, population profiles, identification of spatial clusters of malaria cases, provision of and access to care, and activity level of health infrastructures.

### General Harmonization Strategy

The chosen approach relies on current national health system data reconciliation and does not require any previous system modifications. Such an approach is comparable to the one in Dell’Erba et al [20], which was developed for the domains of travel and tourism information systems and data, or Zinszer et al [21] for malaria data integration. This approach is likely to facilitate the participation of surveillance agencies in the development of a CBMIS, whereas these agencies would be “reluctant to abandon their own data schemata in favor of a standard schema supplied by someone else” [20]. In that sense, the proposed approach differs from recommendations provided in D’Agostino et al [22] to facilitate data sharing in public health, which include the development of regional frameworks that “can be adopted or adapted by each country through national or subnational policies” as a prerequisite for the realization of data interoperability.

In Al Manir et al [23], the authors developed a set of services to query multisource heterogeneous malaria-related data using standard terminologies and rules to match database fields and controlled vocabularies. They illustrated the functioning of the system by answering thematic questions provided by the Uganda Ministry of Health and by querying two data repositories: the Scalable Data Integration for Disease Surveillance platform [21] and the Global Malaria Mapper from the WHO, now integrated into the Global Health Observatory data [24]. The system was not designed to provide and visualize comparable and qualified raw epidemiological data as in this study. However, it can automatically identify any change in source databases and provides tools to reconfigure the system in order to maintain its integrity, unlike our method. Such functionality would be of interest in applying the approach proposed in this article to a large number of surveillance systems.

### Data Completeness, Quality, and Limitations

In French Guiana, CDPSs are not the only malaria notifiers. Nevertheless, given the care pathway of the people living in or frequenting the three border municipalities, the quasi-totality of the malaria cases is retrieved by the system. On the other hand, the three French Guiana border municipalities have only been reporting putative places of infection since 2017, and a lot of missing data are associated with this field. As a consequence, some malaria cases can be omitted by the system if their notifications and places of residence are out of the cross-border area, but the putative places of infection would belong to it. However, we can expect such a number to be negligible. In Brazil, the legal Amazon, whose malaria cases are reported in the SIVEP-Malária, accounts for more than 99% of the Brazilian malaria cases [25,26]. In conclusion, the CBMIS reports reliably on the number of cases within the cross-border area.

Some database attributes exhibit a lot of missing data. Among them, the putative place of contamination, and to a lesser extent the place of residence, is by far the least informed in the CDPS database. However, the information on putative places of contamination has been collected for a long time in French Guiana and has been used for malaria control. The epidemiological bulletins on malaria in French Guiana, published by the national agency for epidemiological surveillance (Santé Publique France), reported that, for the whole French Guiana area and the period between January 2017 and September 2019, the suspected place of contamination is known for 76.9% of cases on average, with a global upward trend (minimum of 54.4% for the first trimester of 2017; maximum of 87% for the first trimester of 2019) (see Multimedia Appendix 1, Table S4). These numbers are comparable with those on the Brazilian side and considerably contrast with those previously shown for French Guiana. In fact, when the CDPS transmits the information on new malaria cases to the local health surveillance authority, the latter requests that the vector control service of the French Guiana territorial collectivity carry out intradomiciliary insecticide spraying and to investigate the context of contamination, in particular, the putative place of contamination. There is currently no back-feeding of the CDPS database with the collected information, which should be considered in the future.

It is worth noting that, despite the difficulties encountered in geocoding all localities on the Brazilian side, the great majority of the new cases reported in Brazil are finally geocoded according to their residence and the place of infection. In fact, only very small localities, and localities that no longer exist, that are associated with very low numbers of cases could not be geocoded. However, efforts are continuing to reach the target of 100% geocoded localities on the Brazilian side.

Some of the missing information in the harmonized database may be due to inadequate coding of the information at the time of notification. However, all possible errors cannot be anticipated and considered within an automatic processing framework unless a highly specific system is built, the functioning of which may become difficult to understand and maintain. The strategy chosen for the CBMIS is instead to provide quality indicators, especially relative to missing information, in order to (1) provide users with the primary interpretation keys in order to let them decide whether an information item is significant or not and (2) give feedback to health actors in charge of surveillance, to allow them to identify surveillance system weaknesses and improve their practice.

The far more difficult point is the interpretation biases derived from differences in country surveillance cultures and practices. Some of these differences are not surmountable, and the harmonization requires making choices and compromises, as with the *new attack* notion discussed above and in Multimedia Appendix 1. Here again, the solution lies in clarifying these differences and the implemented harmonization rules. Multimedia Appendix 1 gathers complementary discussion points that can help inform interpretation of the harmonized data. Eventually, for complementary knowledge on SIVEP-Malária data quality, readers are encouraged to refer to existing publications on the subject [12,27].

### Method Reproducibility

The entire development of the harmonization and visualization applications was carried out with the constant concern that they can be easily and rapidly implemented in other cross-border contexts.

This was ensured by satisfying standards and using existing dedicated and open source tools for data harmonization and visualization. Moreover, the objects of study (ie, patient, consultation, locality, etc) and their properties were formalized by an application knowledge model that currently takes two forms: a dump of the database structure in Structured Query Language (SQL) for its implementation within a database management system such as PostgreSQL, and an ontological formalization in Web Ontology Language (OWL) [28] that enables the knowledge model to be represented according to web data standards and thus ensures its dissemination and reuse by other projects and platforms. Future work will focus on updating and enriching this ontology.

The French Guiana–Brazil cross-border area proved to be an excellent laboratory for the cross-border malaria surveillance issue. It gathers all the specific characteristics of cross-border territories, which make the cross-border malaria issue a major obstacle for the elimination of the disease [2]. The characteristics are as follows: a high diversity of cultures, activities, lifestyles, and languages among the populations; different conceptions, strategies, and means of surveillance, prevention, and control of the disease from one country to another; difficulties in following up with some populations due to their high mobility and possible situations of illegality (ie, undocumented people, illegal activities, etc); and marginalization of border areas with respect to national territorial management and implementation of national public health policies. Moreover, the existing national surveillance systems present significant systemic, syntactic, and semantic differences, and both countries impose different and constraining legal requirements. All the previously listed features make the study area representative of situations we are likely to encounter elsewhere, especially at the international borders of the Brazilian Amazon.

All of the above ensures reproducibility of the method. In fact, the approach was successfully tested at the border between Colombia and Brazil, where a similar monitoring system is currently being developed.

### Cross-Border Malaria Dynamics

Interannual dynamics of malaria case numbers result from a conjunction of multiple factors, and it is difficult to state which one is predominant. However, a few suggestions can be made. Thus, the use of RDTs and the introduction of artemisinin-based combination therapies from 2007 in the CDPSs of French Guiana can explain the drop in cases in French Guiana from 2008 [29]. Moreover, in 2008 with the start of the military operation Harpie, which followed operations Anaconda and Toucan, the French army significantly increased pressure on illegal gold mining in French Guiana, expelling more illegal workers, mainly to Brazil, and tending to make illegal gold mining unprofitable. Although there is a delay of one year, this may partly explain the drop in the number of cases reported in French Guiana from 2009 onward, since the gold-miner population represents one of the major *Plasmodium* species reservoirs in French Guiana [30,31].

In 2012, a binational campaign of distribution of long-lasting insecticide-treated mosquito nets (55 mg/m^2^ concentration of deltamethrin) was carried out on both sides of the French Guiana–Brazil border, co-conducted by the regional health agency of French Guiana (Agence Régionale de Santé de la Guyane) and the health secretariat of the municipality of Oiapoque in Brazil. This may have contributed to the drop in *P falciparum* cases from 2013.

The recrudescence of the case numbers in 2017 and 2018 is more difficult to explain. In fact, such a recrudescence concerned five countries of the Americas according to the Pan American Health Organization [32]: Brazil, Ecuador, Mexico, Nicaragua, and Venezuela. Brazil reported 174,522 cases between January and November 2017 (ie, 56,690 cases more than for the same period in 2016, which represents a 48% increase) [32]. The Amapá state, meanwhile, has seen the number of cases increase by 23%. French Guiana experienced a significant increase of malaria case numbers for the same period, especially in the municipalities at the border with Brazil [33].

The low number of cases in 2019 can be partly explained by concomitant action-research projects, even if their impacts have still to be evaluated. In 2017 and 2018, the ELIMALAR-PALUSTOP (Elimination of Malaria – Stop Paludisme) project performed an active *Plasmodium* species mass screening by molecular biology—polymerase chain reaction method—among 1566 inhabitants of the Saint-Georges municipality, followed by the treatment of all symptomatic and asymptomatic cases. This should have contributed to the decrease of transmission in this cross-border area. In addition, in 2018 and 2019, the French-Brazilian Malakit project distributed self-diagnosis and self-treatment kits to the gold miners in this cross-border area [34].

Differences in follow-up protocols between French Guiana and Brazil can explain the relatively high number of cases associated with follow-up, possible treatment failures, and relapses in French Guiana. The Brazilian health system involves community health workers who visit patients and help with compliance with treatment. On the other hand, in French Guiana, the health system does not benefit from the action of community health workers. Moreover, Brazil systematically gives primaquine to patients with *P vivax*—except for specific cases including pregnancy—which significantly reduces the risk of relapses, whereas prior glucose-6-phosphate dehydrogenase testing is required in French Guiana, which tends to restrict and delay the use of primaquine [33,35]. This situation makes French Guiana more likely to observe *P vivax* relapses than Brazil. In Brazil, patients with good compliance do not experience relapses; in addition, their follow-up does not require consultations at the health centers and does not generate new notifications in the Brazilian system. Eventually, such differences can be explained by the fact that the rule for the non-*P falciparum new case* identification implies a longer delay in French Guiana (90 days) than in Brazil (60 days) (see Methods section and Multimedia Appendix 1).

### International Cooperation

Partnership was a key factor in the success of the CBMIS development. In fact, an operational multilevel—from local health actors to national organizations—and multidisciplinary partnership, including data science, information systems, epidemiology, parasitology, geography, and geomatics, has been strengthening for about eight years within the framework of several research and regional cooperation programs. Such a partnership is able to mobilize skills and know-how to study other cross-border contexts. The co-construction of the system with all partners ensures its appropriation by health actors so that the system can actually enter into the practice of surveillance and ensure targeted and coordinated public health responses from both countries in order to achieve malaria elimination.

### Conclusions

We propose a system that provides comparable and qualified data on the cross-border malaria epidemiological situation. The system is built on technological advances and existing national monitoring systems. Implementing such a system required the application of development good practices, some of which are compulsory, such as those related to privacy, while others contribute to the easy and regular updating of data, facilitate the method’s reproducibility, and ensure confidence in the system, thus ensuring the appropriation of results by user communities.

The resulting system is accessible to territory managers, caregivers, researchers, and the general public. The system can notably help in producing new scientific evidence on disease dynamics and determinants, facilitate cross-border cooperation regarding malaria prevention and control, and contribute to citizens’ informed participation in public debate and in public authority accountability, in order to achieve malaria elimination.

## Appendix

Multimedia Appendix 1 Harmonization rules and algorithm (Tables S1 and S2; Figure S1); online dashboard description (Table S3); and percentage of cases with a specified putative infection location in French Guiana, according to epidemiological bulletins of the interregional epidemiology unit of French Guiana (CIRE [Cellule Inter-Regional d’Epidemiologie; Inter-Regional Epidemiological Center]-Guyane/Santé Publique France) (Table S4).

## Abbreviations

CAPES: Coordenação de Aperfeiçoamento de Pessoal de Nível Superior (Coordination for the Improvement of Higher Education Personnel)
CBMIS: cross-border malaria information system
CDPS: Centre Délocalisé de Prévention et de Soins (Center for Prevention and Care), or Service des Centres Délocalisés de Prévention et de Soins (Department of the Centers for Prevention and Care)
CIRAD: Centre de Coopération Internationale en Recherche Agronomique pour le Développement (French Agricultural Research Centre for International Development)
CNIL: Commission Nationale de l’Informatique et des Libertés (National Commission for Computing and Liberties)
ELIMALAR-PALUSTOP: Elimination of Malaria – Stop Paludisme
ETL: extract, transform, and load
FAPEAM: Fundação de Amparo à Pesquisa do Estado do Amazonas (Amazonas State Research Support Foundation)
FAPEAP: Fundação de Amparo à Pesquisa do Estado do Amapá (Amapá State Research Support Foundation)
FAPEMA: Fundação de Amparo à Pesquisa do Estado do Maranhão (Maranhão State Research Support Foundation)
Fiocruz: Fundação Oswaldo Cruz (Oswaldo Cruz Foundation)
GAPAM-Sentinela: Guyane Française – Amapá – Amazonas – Malária: Sítio Sentinela Transfronteiriça do Observatório Clima e Saúde (French Guiana – Amapá – Amazonas – Malaria: Cross-Border Sentinel Site of the Brazilian Climate and Health Observatory)
IRD: Institut de Recherche pour le Développement (French National Research Institute for Sustainable Development)
LMI: Laboratoire Mixte International (Joint International Laboratory)
LVC: lâmina de verificação de cura (treatment verification slide)
ODYSSEA: Observatory of the Dynamics of Interactions Between Societies and Environment in the Amazon
OWL: Web Ontology Language
PrInt: Programa de Internacionalização (Internationalization Program)
RDT: rapid diagnostic test
SIVEP-Malária: Sistema de Informações de Vigilância Epidemiológica da Malária (Malaria Epidemiological Surveillance Information System)
SQL: Structured Query Language
WHO: World Health Organization
