# Supplementary material for: Contributing to Elimination of Cross-Border Malaria Through a Standardized Solution for Case Surveillance, Data Sharing, and Data Interpretation: Development of a Cross-Border Monitoring System
Source: JMIR Public Health Surveill. 2020 Sep 1;6(3):e15409. doi: 10.2196/15409 (PMC7492983; doi:10.2196/15409)
Supplement: Multimedia Appendix 1 [file publichealth_v6i3e15409_app1.pdf]

Table S1: Information retrieved from the French and the Brazilian databases, maintained by the Delocalized Centers for Prevention and Care (CDPS) and the malaria epidemiological surveillance information system (SIVEP-Malária), respectively, and description of the harmonized attributes and the harmonization rules. Notations "n/s" and "n/a" are for "not specified" and "not applicable", respectively. For the complete list and description of the SIVEP-Malária attributes, see http://www.consultaesic.cgu.gov.br/busca/dados/Lists/Pedido/Attachments/689497/RESPOSTA_PEDIDO_Dicionrio%20de%20dados-%20Sivep-Malria.pdf

Information type	Attribute name (attribute type): domain of values, source or format		Harmonized field name (type) : domain of values, source or format	Applied standard for harmonized data	Harmonization rules and comments
	Brazil	French Guiana			
Unique patient identifier	n/s	NumeroPatient (character string)	n/a	n/a	At the launch of the CDPS surveillance system in 2007, patient identifiers were very often not entered properly. Until 2013, identifiers of dead patients were sometimes reassigned to new patients. This leads to an unreliable determination of patient history based on his/her identifier, and possibly erroneous classification of the patient notifications as "new case", "follow-up" or "relapse or treatment failure". In 2013, such a practice was prohibited, but possible remaining cases could have persisted during the two following years, from 2013 to 2015.
Administrative unit of origin of the data	n/a	n/a	source (character string): FR-GF: French Guiana; BR: Brazil	ISO 3166-2	
Sex	SEXO (character string): M: Male; F: Female; I: Unknown	SexePatient (integer): 1: Male; 2: Female; 0: Unknown	patient_sex (integer): 1:Male; 2:Female	ISO/CEI 5218	Conversion from character string to integer for Brazilian data.
Age	ID_PACIE (integer)	AgePatient (real): years	patient_age (real): age in years		Age conversion from day or month to year for Brazilian data.
Age value unit	ID_DIMEA (character string): D: dias; M, Months; A Years	n/s	n/a	n/a	

Information type	Attribute name (attribute type): domain of values, source or format		Harmonized field name (type) : domain of values, source or format	Applied standard for harmonized data	Harmonization rules and comments
	Brazil	French Guiana			
Notification identifier	COD_NOTI (integer):	n/s	id_consultation (character string)	n/a	• Sequential identifier for French Guiana;<br>• As SIVEP-Malária notification codes are not unique in the SIVEP-Malária, unique notification codes were formed by concatenating the initial notification code, the notification date and the notification centre code (e.g. 1_Fri Apr 29 00:00:00 GFT 2005_128)
Notification date	DT_NOTIF (date): dd/mm/YYYY	DateConsultation (date): dd/mm/YYYY	consultation_date (date): dd/mm/YYYY	ISO 8601	
Health unit identifier	COD_UNIN (integer): SIVEP-Malária codes	CodeCS (character string): CDPS code	id_center (character string) : CDPS and modified (see comments) SIVEP-Malária codes	SIVEP-Malária and CDPS specific codes for, respectively, Brazil and French Guiana	• Residence and infection locations are chosen in the same location list (one list per country);<br>• As SIVEP-Malária locality codes (resp. notification center codes) are unique at the municipality level (i.e., two locations - resp. two notification centers - from two different municipalities can have the same code), Brazilian locality codes (resp. notification center codes) were formed by concatenating locality (resp. notification center) and municipality codes (e.g. 87/160050 for the Kumenê locality; 128/160050 for the Planalto health center);<br>• Information on the possible locality of infection is only collected from 2017 in French Guiana.
Locality of residence	LOC_RESI (integer): SIVEP-Malária codes	ZoneHabitationPatient (character string): CDPS codes	residence_place (character string): CDPS and modified (see comments) SIVEP-Malária codes		
Possible locality of infection	LOC_INFE (integer): SIVEP-Malária codes	LieuContamination (character string): CDPS codes	infection_place (character string): CDPS and modified (see comments) SIVEP-Malária codes		
Municipality of residence	MUN_RESI (integer): IBGE code	n/s	residence_municipality (integer): e.g., 160050: Oiapoque; 97308: Saint-Georges-de-l'Oyapock, etc.	INSEE and IBGE codes for, respectively, French and Brazilian municipalities	CDPS recruiting zones can correspond to cross-border territories. Consequently, the following rules were defined for French data: IF ZoneHabitationPatient (resp. LieuContamination) is EMPTY THEN residence_municipality (resp. infection_municipality) is set to EMPTY[1], ELSE (IF ZoneHabitationPatient (resp. LieuContamination) ∈ {ESGOX_VICT[2], ESGOX_OIAP[3], ECAMX_BRAZ[4]} THEN residence_municipality (resp. infection_municipality)=160050[5], ELSE residence_municipality (resp. infection_municipality)=French municipality associated with the recruitment zone).
CDPS zone of recruitment where the patient lives	n/s	ZoneRecrutement (character string): CDPS codes			
Municipality of contamination	MUN_INFE (integer): IBGE code	n/s	infection_municipality (integer): e.g., 160050:	INSEE and IBGE codes for, respectively, French and	

Information type	Attribute name (attribute type): domain of values, source or format		Harmonized field name (type) : domain of values, source or format	Applied standard for harmonized data	Harmonization rules and comments
	Brazil	French Guiana			
CDPS zone of recruitment where the patient was possibly contaminated	n/s	ZoneContamination (character string): CDPS codes	Oiapoque; 97308: Saint-Georges-de-l'Oyapock, etc.	Brazilian municipalities	(1) A significant part of the cases notified in French border municipalities can reside in Brazil. Without information on the residence location, the recruitment zone is not reliable information for the determination of the residence municipality<br>(2) Vila Vitória and Santo Antônio<br>(3) Oiapoque city<br>(4) Vila Brasil<br>(5) Oiapoque municipality
State of residence	UF_RESID (integer): IBGE codes	n/s	residence_state (character string): e.g., BR-AP: Amapá; BR-PA: Pará; FR-GF: French Guiana, etc.	ISO 3166-2	• Concerning French data, rules comparable to those for residence and infection municipalities are applied;<br>• French Guiana is a French territorial community and is not strictly-speaking a "state". However, it can be compared to Amapá concerning the administrative organization of France and Brazil.
State of contamination	UF_INFEC (integer): IBGE codes	n/s	infection_state (character string): e.g., BR-AP: Amapá; BR-PA: Pará; FR-GF: French Guiana, etc.		
Country of residence	PAIS_RES (integer): IBGE codes	n/s	residence_country (character string): BR: Brazil; FR: France	ISO 3166-2	
Country of infection	PAIS_INF (IBGE code): IBGE codes	n/s	infection_country (character string): BR: Brazil; FR: France		
Type of diagnosis (active or passive)	TIPO_LAM (integer): 1: passive detection; 2: active detection; 3: treatment control slide (Lamina de Verificação de Cura, LVC)	n/a	active_diagnosis (boolean): TRUE or FALSE	n/a	• Until now, CDPS only perform passive diagnosis. However, possible changes can occur in the future, and we chose to keep this attribute in the harmonized database;<br>• In the harmonized database, treatment control slides (Lâmina de Verificação de Cura, LVC) are considered as passive detection.

Information type	Attribute name (attribute type): domain of values, source or format		Harmonized field name (type) : domain of values, source or format	Applied standard for harmonized data	Harmonization rules and comments
	Brazil	French Guiana			
Plasmodium species	RES_EXAM (integer):<br>1: None<br>2: *P. falciparum*<br>3: *P. falciparum* + *P. falciparum* gametocytes<br>4: *P. vivax*<br>5: *P. falciparum* + *P. vivax*<br>6: *P. vivax* + *P. falciparum* gametocytes<br>7: *P. falciparum* gametocytes<br>8: *P. malariae*<br>9: *P. falciparum* + *P. malariae*<br>10: *P. ovale*<br>11: other than *P. falciparum*	DiagnosticPrincipal and AutreDiagnostic (character string): International Classification of Diseases, 10th Revision (ICD10)<br><br>and<br>PaluFalci<br>and<br>PaluAutre (boolean): TRUE, FALSE	diagnosis_result (character string): P. *falciparum*; *non-falciparum*; Mixed infections with *P; falciparum*; Unspecified)	n/a	• See Supplementary Table S2 for the coding of the Brazilian information;<br>• For French Guiana, the information on the *Plasmodium* species can be present in 4 different fields of the CDPS database. These fields can bring redundant information (e.g., diagnosticprincipal="B51" and paluautre=TRUE) or complementary one (e.g. diagnosticprincipal="B54" and palufalci=TRUE). All this information is combined to determine the *Plasmodium* species;<br>• Some specific coding errors in the CDPS database are taken into account. For example, "palu vivax" mentions (wrong code for a *P. vivax* case that should be coded "B51") are identified and coded as non-falciparum in the harmonized database;<br>• ICD10 could appear to be a standard for diagnosis result coding, but in fact, it is not directly applicable to the result of the Rapid Diagnosis Tests (e.g., non-falciparum would correspond to "B51" or "B52" or "B53" code) ;<br>• The term "non-*P. falciparum*" was chosen in order to be in complete agreement with the information provided by the RDT. However, *P. vivax* represents the vast majority of the non-*P. falciparum* cases in the study region, *P. malariae* representing only 0.2% of the cases notified in the Oiapoque municipality between 2003 and 2015 [8].
New infection vs. notification related to patient's follow-up, treatment failure or relapse	ID_LVC (integer): 1: *is* a treatment verification slide; 2: is *not* a treatment verification slide	n/s	new_attack (boolean): TRUE, FALSE	n/a	See article and supplementary file S3 for details

Table S2: Correspondence between SIVEP-Malária and harmonized database codes for the *Plasmodium* species

**SIVEP-Malaria database**		**Harmonized database**
**Attribute: RES_EXAM**	**Descri ption**	**Attribute: diagnosis_result**
1	Negative	*not considered (all cases in the harmonized database are positive)*
2	*P. falciparum*	*falciparum*
3	*P. falciparum* and *P. falciparum* gametocytes	*falciparum*
4	*P. vivax*	non-*falciparum(*)*
5	*P. falciparum* and *P. vivax*	mixed infection with *P. falciparum*
6	*P. vivax* and *P. falciparum* gametocytes	mixed infection with *P. falciparum*
7	*P. falciparum* gametocytes	*falciparum*
8	*P. malariae*	non-*falciparum*
9	*P. falciparum* and *P. malariae*	mixed infection with *P. falciparum*
10	*P. ovale*	non-*falciparum*
11	non-*P. falciparum*	non-*falciparum*

(*) "non-*P. falciparum*" was chosen in order to be in complete agreement with the information provided by the RDT. However, *P. vivax* represents the vast majority of the non-*P. falciparum* cases in the study region. *Plasmodium malariae* is present but rare (Musset et al., 2014), representing only 0.2% of the cases notified in the Oiapoque municipality between 2003 and 2015 (da Cruz Franco et al., 2019).

References :

Musset L, Pelleau S, Girod R, Ardillon V, Carvalho L, Dusfour I, Gomes M, Djossou F, Legrand E. Malaria on the Guiana Shield: a review of the situation in French Guiana. Memórias do Instituto Oswaldo Cruz. 2014;109:525–33. PMID:25184998

da Cruz Franco V, Peiter PC, Carvajal-Cortés JJ, dos Santos Pereira R, Mendonça Gomes M do S, Suárez-Mutis MC. Complex malaria epidemiology in an international border area between Brazil and French Guiana: challenges for elimination. Trop Med Health 2019;47:1-12. PMID:31007535

Table S3: Main features of the two versions of the online dashboards

<table>
<tr><th>Feature</th><th>General Public version</th><th>Expert version</th></tr>
<tr><td>URL</td><td>https://shiny.icict.fiocruz.br/publicirdmalaria/</td><td>https://irdmalaria.icict.fiocruz.br</td></tr>
<tr><td>Authentification</td><td>None</td><td>Yes (Login/Password)</td></tr>
<tr><td rowspan="6">Tabs</td><td colspan="2">“Harmonized indicators”: malaria case numbers time-series and bar charts on age and sex case distribution, by countries</td></tr>
<tr><td colspan="2">“By location”: time-series by localities (notification centers, patient residence or putative infection place)</td></tr>
<tr><td colspan="2">“Ranking”: Ranking of localities by case numbers</td></tr>
<tr><td colspan="2">“Maps”: Map</td></tr>
<tr><td></td><td>“Information quality”: missing information per variable</td></tr>
<tr><td></td><td>“Dowload”: dowloadable data table</td></tr>
<tr><td rowspan="8">Filters on data</td><td colspan="2">Time aggregation by months, quarters, years</td></tr>
<tr><td></td><td>Daily and weekly time aggregation</td></tr>
<tr><td colspan="2">Period of visualization</td></tr>
<tr><td colspan="2">Patient gender</td></tr>
<tr><td colspan="2">Patient age</td></tr>
<tr><td colspan="2">Plasmodium species</td></tr>
<tr><td colspan="2">Case status (new attack, not new case, i.e. follow-up, treatment failure or relapses, any case status)</td></tr>
<tr><td colspan="2">Detection method (active or passive)</td></tr>
</table>

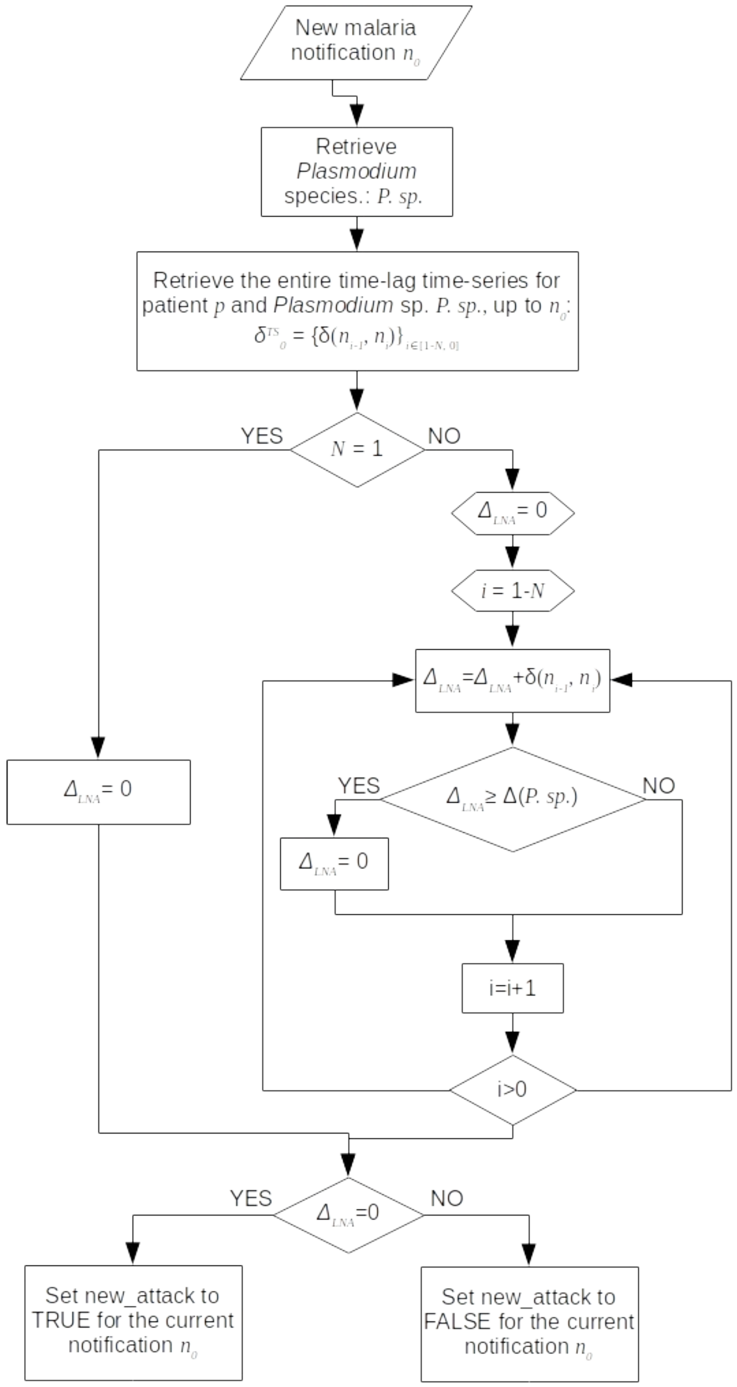

Figure S1: Algorithm permitting to qualify a French Guiana case notification as a "new attack" of malaria, *i.e.* a new infection due to an infected mosquito bite (new_attack=TRUE), or a notification associated to the patient follow-up protocol, a suspected relapse (*P. vivax*) or a treatment failure (*P. vivax* and *P. falciparum*) (new_attack=FALSE).

P. sp. is for the *Plasmodium* species, and Δ(*P. sp.*) returns 40 or 90 days depending on whether *P. sp.*=*P. falciparum* or *P. sp.*=non-*P. falciparum*, respectively; $\delta(n_i, n_j)$ returns the time lag between the notification *i* and *j*; δ^{TS}_i is the time-series of the time lags between the successive notifications, until the notification *i*; *N* is the length of δ^{TS}_0, *i.e.*, the total number of notifications of the patient; Δ_{LNA} is the time lag between the current notification and the Last New Attack (LNA).

NB: The satisfaction of legal and ethical rules (see Article) complicated the harmonization process. Notably, the fact that the unique patient identifier (French side), considered as personal data, can only be kept for a limited time (one year in our case) complicated the implementation of the rules for detecting new attacks. The solution found to be able to retrospectively apply different detection rules in the absence of patient identifiers was to store, for each notification and by considering the two *Plasmodium* species (non-*P. falciparum* and *P. falciparum*) separately, the time series of the intervals between the successive notifications of the patient.

New attacks of *P. vivax* are not detected with the same rule in Brazil and French Guiana. Detection rules were chosen to realize the best compromise between parasitology knowledge status and actual epidemiologists practices in both countries (see article main text). However, applying the same detection rule would not be necessarily justified because the treatment protocol for *P. vivax* malaria cases differ in French Guiana and Brazil. Brazil systematically gives primaquine to patients with *P. vivax* malaria (except for specific cases including pregnancy), which significantly reduces the risk of relapses, whereas French Guiana conditions its use to prior G6PD testing. Consequently, French Guiana is likely to observe more *P. vivax* relapses than Brazil.

Table S4: Percentage of cases with an specified putative infection place in French Guiana, according to epidemiological bulletins of the inter-regional epidemiology unit of French Guiana (CIRE-Guyane/Santé Publique France).

Period		Percentage of cases with an informed probable infection place	Volume of the epidemiological bulletin	Links to the epidemiological bulletins
Beginning	End			
01/2017	03/2017	54.4	2017(02)	https://www.santepubliquefrance.fr/content/download/155593/2210102
04/2017	06/2017	74.2	2017(03)	https://www.santepubliquefrance.fr/content/download/40666/817432
07/2017	09/2017	75.5	2017(04)	https://www.santepubliquefrance.fr/content/download/40213/802849
09/2017	11/2017	82.5	2017(05)	https://www.santepubliquefrance.fr/content/download/40031/797135
11/2017	01/2018	75.4	2018(01)	https://www.santepubliquefrance.fr/content/download/39508/780518
01/2018	06/2018	82.0	2018(02)	https://www.santepubliquefrance.fr/content/download/38676/754437
07/2018	11/2018	80.0	2018(03)	https://www.santepubliquefrance.fr/content/download/38105/736216
01/2019	03/2019	87.0	2019(01)	https://www.santepubliquefrance.fr/content/download/132096/2016732
04/2019	09/2019	81.1	2019(02)	https://www.santepubliquefrance.fr/content/download/204185/2393033
